# Emerging Concepts on Infection of Novel Cardiac Implantable Devices

**DOI:** 10.31083/j.rcm2308277

**Published:** 2022-08-05

**Authors:** Mohammad Said Ramadan, Raffaella Gallo, Fabian Patauner, Lorenzo Bertolino, Emanuele Durante-Mangoni

**Affiliations:** ^1^Department of Precision Medicine, University of Campania ‘L. Vanvitelli’ Napoli, Italy and Unit of Infectious and Transplant Medicine, AORN Ospedali dei Colli-Monaldi Hospital, 80131 Napoli, Italy

**Keywords:** new cardiac device, infection, infective endocarditis, Micra, MitraClip, occluder devices, cardiac contractility modulation

## Abstract

Novel cardiac devices, including the MitraClip system, occluder devices, leadless pacemakers, and subcutaneous implantable cardioverter defibrillators (S-ICD), are mostly used in the management of patients who are at high risk for surgery and/or developing infections. Several mechanisms render most of these devices resistant to infection, including avoiding long transvenous access and novel manufacturing material. Since subjects who use these devices already endure several comorbid conditions, uncommon cases of device-associated infection could result in serious complications and increased mortality. In this review, we aim to summarize the current state of evidence on the incidence, clinical presentation, management, and prognosis of new cardiac devices’ associated infection.

## 1. Introduction

Heart failure currently affects 3–4% of the worldwide population with a prevalence peaking up to 10% after 70 years of age and a constant rise in incidence [1]. Such heart failure epidemic has vigorously stimulated the field of interventional cardiology and boosted the development and widespread use of a number of newer cardiac devices, meant to correct structural or functional cardiac defects. Novel cardiac devices such as MitraClip system (Abbott Vascular, Santa Clara, CA, USA), left atrium appendage occluder devices, septal closure devices, leadless pacemakers (LP), and subcutaneous implantable cardioverter defibrillators (S-ICD), provide safe treatment options for heart failure patients deemed at high-risk for more invasive procedures or open-heart surgery [2, 3, 4, 5, 6]. Unique mechanisms allow for these devices to be less prone to infections, including avoiding of long electronic leads, intravenous access, subcutaneous pockets, or large prostheses and use of novel device materials, as compared to traditional transvenous implantable electronic devices and valve prostheses.

Although supposedly less common, infective endocarditis (IE) involving these newer devices occurs. In light of the steep increase in implantation rates, it appears crucial to properly describe new device-associated infections, and understand the associated burden and whether heightened morbidity and mortality should be expected.

In this review, we aim to summarize the current published evidence on the incidence, clinical characteristics, management, and outcomes of patients who developed IE following implantation of the newer implantable cardiac devices.

## 2. MitraClip 

**Fig. 1.**
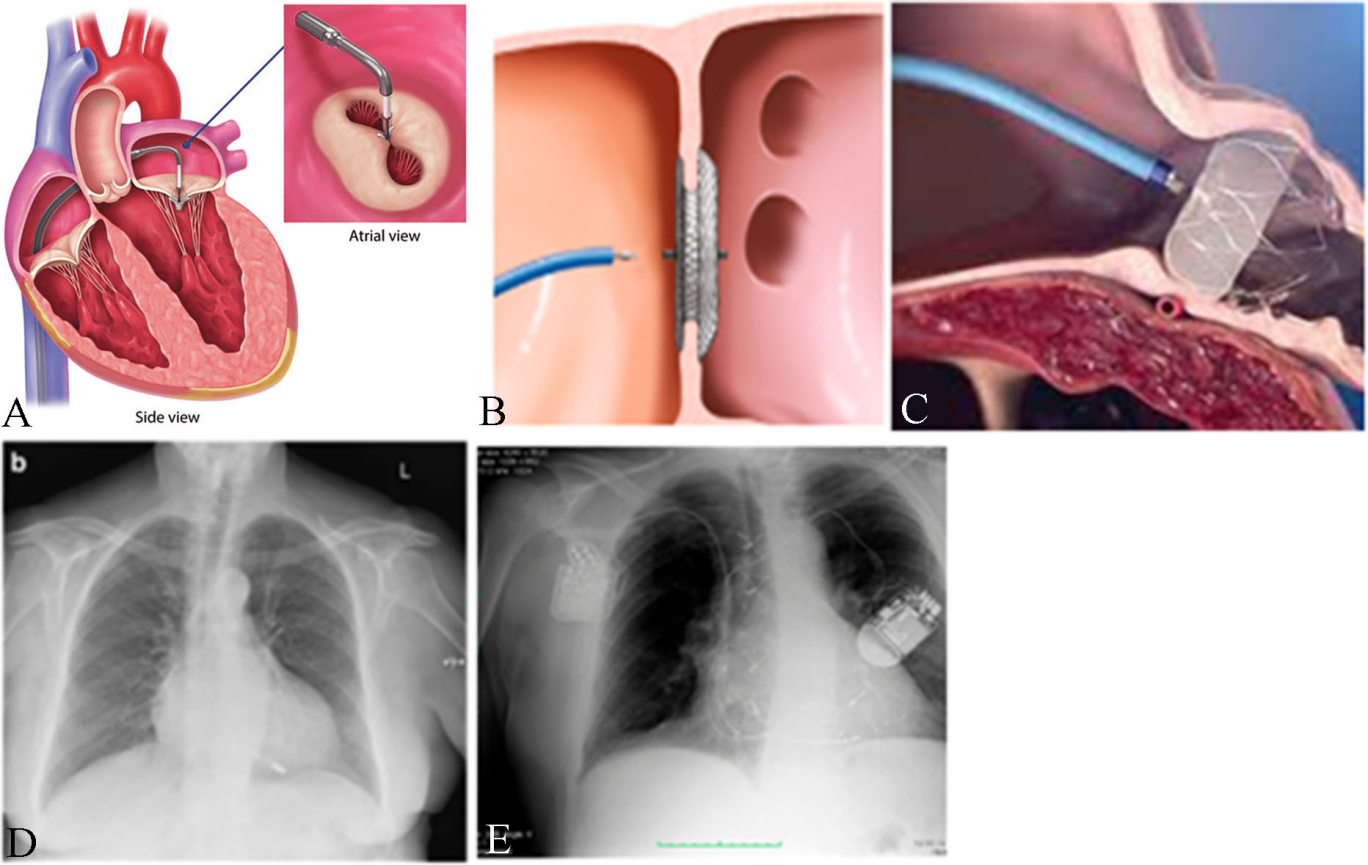
**Novel Cardiac Devices**. (A) MitraClip system [9] (via license: Creative Commons Attribution 3.0). (B) Amplatzer® ASD closure device [10]. (C) WATCHMAN LAAO device [11]. (D) Micra® (adapted from [12], Creative Commons Attribution 4.0 International License). (E) Cardiac contractility modulation device (right side) (adapted from [13], Creative Commons Attribution 4.0 International License). ASD, atrial septal defect; LAAO, left atrial appendage occlude.

MitraClip is a percutaneously implanted cardiac device that allows treatment of severe mitral regurgitation (MR) in patients with annular dilatation and chordal stretching due to dilated cardiomyopathy, and therefore at high risk for open heart surgery [7, 8]. It essentially replicates the edge-to-edge mitral valve repair strategy but with a percutaneous and noninvasive approach (Fig. 1A, Ref. [9, 10, 11, 12, 13]).

Incidence of infective endocarditis (IE) following MitraClip was investigated by several studies [6, 14]. In the pivotal EVEREST II trial [14], where patients were followed up for 12 months, 1.1% (2/184 pts) had IE throughout the study period. This was confirmed in subsequent studies which found the infection risk to range between 0 and 1.3% (75% within 1-year after procedure) [6]. However, the exact incidence of MitraClip-associated IE remains unclear and could likely increase in parallel with the rising rates of procedure performance.

The first reported case of MitraClip-associated IE [15], published in 2011, was a 57 year-old man with early onset IE (2 weeks after device implantation) diagnosed by blood cultures positive for *Staphylococcus aureus* and a mobile lesion on the MitraClip seen on transthoracic (TTE) and transesophageal echocardiography (TEE). Management included targeted antibiotics and, despite the high predicted perioperative mortality (EuroSCORE II 18%), surgical mitral valve replacement, which histologically confirmed IE. Few years later, Frerker *et al*. [16] described the case of an 88 year-old patient, with high surgical risk (EuroSCORE II 30.4%) and severe MR, presenting with fever and shortness of breath, one month after MitraClip placement. Echocardiography showed large vegetations on the mitral valve within the clip area, complicated by severe MR, and serial blood cultures were positive for *S. aureus*. Therefore, definite early IE was diagnosed according to the modified Duke criteria [17]. The patient underwent cardiac surgery (logistic EuroSCORE 56.8%) with mitral valve replacement and discharged alive to a rehabilitation facility.

In a review of 12 cases, Asmarats *et al*. [6] showed that patients with MitraClip-related IE are usually older (median age 76 years) than patients with valvular IE (median age 62 years) [18], and present a higher burden of comorbidities [19]. Indeed, this procedure is indicated in patients at high preoperative surgical risk. Undoubtedly, the mentioned clinical features influence the high mortality associated with MitraClip-related IE, as they are reported to be among the predictors of poor outcome [20]. Upon pooling of available literature data, we were able to appreciate that the clinical presentation of MitraClip-related IE is dominated by classical symptoms of systemic infection (fever in 15/25 pts; 60%) together with signs and symptoms of decompensated heart failure (12/25 pts; 48%) [6, 15, 16, 21, 22, 23, 24, 25, 26], resulting from mitral valve recurrent regurgitation or stenosis. Less common presentations included complete heart block (one case) [27], whilst septic emboli seldom occurred (3/25 pts; 12%) [28, 29].

MitraClip-related IE occurred early (<12 months) in most reported cases (18/25 pts, 72%; 75% according to Asmarats *et al*. [6]). This suggests that the infection could be often acquired perioperatively. Indeed, it has been demonstrated that implanted MitraClips go through a physiological healing process of fibrous encapsulation that completes within around 300 days [30, 31], after which the risk of pathogens’ seeding appears to be significantly reduced.

Diagnostic criteria do not differ from those currently employed for valvular IE [17] and are based on echocardiography findings and blood cultures. However, alternative imaging technique as ^18^F-fluorodeoxyglucose PET/CT could be appropriate in selected cases [29]. *S. aureus* is the major causative microorganism (11/25 pts; 44%). However, infections of MitraClip caused by *Enterococci* (3/25 pts, 12%), *Streptococci* (2/25 pts, 8%) [6, 32], *Pseudomonas aeruginosa* [33] or atypical microorganisms such as *Bartonella haenselae * [34] are also reported.

Treatment of MitraClip IE is challenging and still debated. Surgical mitral valve replacement was needed in 56% of patients despite the extremely high calculated preoperative mortality risk (mean logistic EuroSCORE of 41% [6]). A conservative approach, based on antibiotic therapy only, can be considered in selected patients; especially those with coagulase negative staphylococcal etiology [35]. The mortality associated to the IE episode is around 52%, higher than prosthetic valve IE (20–40%) [20].

In conclusion, MitraClip-associated IE is a very uncommon but potentially fatal complication affecting patients with advanced age and multiple comorbidities. It often requires surgical treatment, but the optimal approach is still unclear and needs to be devised on an individual basis.

## 3. Closure Devices and Shunts

Closure or Occluder devices are an increasingly implanted class of devices [36]. These include devices implanted to close atrial or ventricular septal defects (Fig. 1B) and devices used to occlude the left atrium appendage in patients with atrial fibrillation (Fig. 1C).

### 3.1 ASD Closure Devices

The three main devices used for atrial septal defect (ASD) and patent foramen ovale (PFO) closure are: Amplatzer® Septal Occluder (ASO, most common, St. Jude Medical, St. Paul Minnesota, USA), Gore Helex® Septal Occluder (HSO, W.L. Gore and Associates, Flagstaff, Arizona, USA), and CardioSEAL- STARFlex® (CardioSEAL; NMT Medical, Boston, Massachusetts, USA) device [37].

After extensive literature review, we found 23 reported cases of IE on ASD closure devices (Table 1, Ref. [38, 39, 40, 41, 42, 43, 44, 45, 46, 47, 48, 49, 50, 51, 52, 53, 54, 55, 56, 57, 58, 59]).

**Table 1.** **Summary of reported cases of ASD closure device-related IE**.

Ref.	Name of device	ASD cases	PFO cases	Presentation	Therapy	Outcome
[38, 39, 40, 41, 42, 43, 44, 45, 46, 47, 48, 49, 50, 51, 52, 53, 54]	ASO	14	3	Septic shock, fever, myalgia, Janeway lesions, ischemic stroke, meningitis, pharyngitis, peripheral emboli, septic arthritis	15/17: Surgery	Cured, discharged alive
					2/17: medical therapy	
[55]	ASDOS	1	1	Pneumonia, pleural effusion, peripheral embolism	Surgery	Dead
[56]	HSO	-	1	Fever, chills	Medical therapy	Complications: cerebral and pulmonary embolisms, discharged alive
[57]	FSO	1	-	Fever, arthralgias, headache, an Osler node, Janeway lesions, cerebral infarct	Surgery: ASD repair	Cured, discharged alive
[58]	STARFlex	-	1	Palpitations	Surgery	Cured, discharged alive
[59]	CardioSEAL	-	1	Fever, throat pain	Surgery	Cured, discharged alive

ASD, Atrial septal defect; PFO, patent foramen ovale; ASO, Amplatzer® Septal Occluder; ASDOS, atrial septal defect occluder system; HSO, Helex® Septal Occluder.

Median age of subjects with device IE was 37 years and most developed endocarditis beyond 6 months of device implantation (17/23, 78%). Fever was the most common presenting symptom (n = 22/23, 95%), and other presentations included stroke/other site embolism (n = 10/23, 42%, pulmonary embolism in one case [56]), septic shock (n = 2/23, 8%), immunologic phenomena (n = 3/23, 13%), and acute meningitis (n = 1/23, 4%).

Identified risk factors for IE in this population included intravenous illicit drug abuse (n = 2/23, 8%), fever before implantation (n = 1, 4%), diabetic foot and osteomyelitis (n = 1/23, 4%), dental care (2/23, 8%), and nasal trauma (n = 1/23, 4%).

Etiological diagnosis of IE was mostly reached by blood cultures (21/23, 91%) and in one case the bacterium was also isolated from synovial fluid. *S. aureus *was detected in more than half of patients (total: 12/23, 52%; methicillin-sensitive: 8/23, 35%; methicillin-resistant: 4/23, 17%), other organisms included other *Staphylococcus *species (2/23, 8.7%) and *Streptococcus pyogenes *(n = 2/23, 8%). Imaging diagnosis was made by TEE in all patients, where the vegetation was more frequently identified on the left atrial side of the device (n = 15/23, 65%), than the right atrial side (n = 2/23, 8%), and one patient also had tricuspid valve involvement (4%). Three patients (3/23, 13%) had vegetations on both left and right sides of the device. The majority of cases (20/23, 87%) were treated with antibiotics, surgical removal of the device, and pericardial patch, whereas the remaining (3/23, 13%) were treated only with antibiotics (of whom two were cured and one died after pulmonary and cerebral embolisms). After removal, incomplete device endothelialization was found in as many as 9 cases (9/20, 45%).

Most patients were discharged alive (21/23, 91%) with no major ongoing complications. However, one patient developed a third degree atrio-ventricular block with need of pacemaker implantation and left hemianopsia, and another developed pulmonary and cerebral embolism (treated only with medical therapy). The remaining two patients (8%) died after surgical removal of the device—which was an atrial septal defect occluder system (ASDOS) in both instances. Causes of death in these patients included sternal wound infection and failure of weaning off extracorporeal circulation, respectively.

In conclusion, despite having a safe profile, ASD and PFO closure devices can become infected. As clinical signs are non-specific, IE should be considered in patients with signs of systemic infection, and diagnosis can be established by blood cultures and TEE. Removal of the device and timely initiation of antibiotic therapy are the mainstays of treatment with close follow-up and monitoring for possible embolic complications.

### 3.2 VSD Closure Devices

Percutaneous closure of ventricular septal defects (VSD) is an alternative to surgery in cases of poor surgical fitness, residual VSDs and muscular VSDs located in the mid part of the interventricular septum [4].

Six cases of VSD closure device-related infective endocarditis (IE) have been reported in the literature so far (Table 2) [4, 60, 61, 62, 63, 64], which included the Amplatzer® device (2/6, 33%), Nit-Occlud® (3/6, 50%), and modified symmetric double-disk occluder (SHAMA) (1/6, 16%). VSD-related IE is mostly a pediatric condition. Median age at IE presentation in these patients was 3.5 years (range: 1.8–15 years) and all presented with fever. Other specific manifestations were not described.

**Table 2.** **Clinical findings in 6 reported cases of VSD closure device-related IE**.

Device	Presentation	Microorganism	Treatment	Outcome	Citation
Amplatzer®	Fever and sepsis	Klebsiella denitrificans	Medical	Cured, discharged alive	[4]
Amplatzer®	Fever	Candida albicans	Surgery, pericardial patch	Cured, discharged alive	[61]
Nit-Occlud®	Fever	Pseudomonas aeruginosa	Surgery	Dead	[63]
Nit-Occlud®	Fever, chills, diarrhea	Kingella kingae	Surgery, pericardial patch	Cured, discharged alive	[60]
SHAMA	Fever	Staphylococcus aureus	Medical	Cured, discharged alive	[62]
Nit-Occlud®	Fever	Staphylococcus aureus	Surgery	Cured, discharged alive	[64]

Most patients developed VSD-related endocarditis early after implantation (5/6, 83%), while the remaining case developed IE 11 years after the procedure. Bacterial identification was achieved by blood culture in all cases, and a range of organisms were isolated including *S. aureus* (*2 cases*), *Klebsiella denitrificans*, *Kingella kingae*, *Candida albicans*, and *Pseudomonas aeruginosa* (1 case each). Imaging diagnosis was achieved by TEE in half of the patients and by TTE only in the remainder; concomitant tricuspid valve involvement was found in two patients (33%).

Treatment included antibiotics, surgical removal of the device, and pericardial patch implantation in 4 patients (66%), tricuspid valve replacement in 2 patients (33%), and antibiotics without surgery in 2 patients (33%).

Five patients (83%) were discharged home alive and in good clinical conditions, while one patient (16%) died after surgical removal of the device, due to progression of heart failure and sepsis (caused by *Pseudomonas aeruginosa*).

### 3.3 Left Atrial Appendage Occluder Devices

One goal of atrial fibrillation (AF) treatment is to prevent formation and systemic embolization of thrombi [65], which are formed in the left atrial appendage in more than 90% of patients with nonvalvular AF associated thrombi [66]. In specific circumstances such as with prior life-threatening hemorrhage or contraindications to long term oral anticoagulants, Left Atrial Appendage Occluder (LAAO) devices are the mainstay of treatment for AF-related embolism [67]. The most widely used LAAO - the WATCHMAN device (Boston Scientific, Marlborough, MA, USA)—was found to be non-inferior to oral anticoagulant therapy in the prevention of stroke, systemic embolism, and cardiovascular death [68, 69, 70], and with very low risk of infection, even in patients with BSI [71].

The WATCHMAN is a novel device used in LAA closure and is a self-expanding nitinol frame structure with fixation barbs and a permeable polyester fabric that covers the left atrial facing surface of the device (Fig. 1C) [72].

Five cases of LAA closure device endocarditis have been reported in the literature to date (Table 3) [73, 74, 75, 76, 77]. Median age of these cases was 74 years (range: 70–83) and onset of the infection was at a median of 140 days (range 6–900 days) after implantation. Presenting signs and symptoms in these patients mostly included fever (4/5, 80%) and fatigue (2/5, 40%). Other symptoms such as chills (1/5, 20%) and neurologic manifestations like confusion (1/5, 20%), neck stiffness (1/5, 20%), and left sided hemiparesis (1/5, 20%), also occurred. In the latter patient, neurologic manifestations were a consequence of cerebral embolism, whereas another patient had detectable although asymptomatic cerebral emboli. One patient also received a diagnosis of osteomyelitis of the great toe [77].

**Table 3.** **Outline of reported cases of LAAO device-related IE**.

Presentation	Micro-organism	Treatment	Outcome	Citation
Leukocytosis	MSSA	Surgery: extraction and then LAA ligation	Brainstem stroke (residual generalized weakness and disability), discharged alive	[73]
Fever, confusion, neck stiffness, left side hemiparesis, endophthalmitis with purulent secretions	Pseudomonas aeuroginosa	Surgery: extraction, implantation of mitral biological prosthesis	Dead	[74]
Fatigue, myalgias, fever, and cough	MSSA	Medical therapy	Cured, discharged alive	[76]
Fever, chills	Enterococcus spp and Enterobacter spp	Surgery: extraction and then LAA ligation	Hemothorax, respiratory failure (requiring tracheostomy), encephalopathy, and right axillary vein thrombosis.	[75]
			Cured, discharged alive	
Malaise, fatigue, an unwitnessed mechanical fall, acute on chronic right foot pain	MRSA and Pseudomonas aeuroginosa	Medical	Cured, discharged alive	[77]

LAA, left atrial appendage; MSSA, methicillin sensitive *S. aureus*; MRSA, methicillin resistant S. aureus.

Diagnosis was made in all cases using TEE and blood cultures; the latter isolated *S. aureus *in most cases (total: 3/5, 60%; MSSA, MRSA, and undefined: 1/5, 20% each, respectively), and other causative organisms included *Pseudomonas aeruginosa *(1/5, 20%, also detected in cerebrospinal fluid culture in this patient), and a polymicrobial culture of *Enterococcus spp and Enterobacter spp* (1/5, 20%).

Treatment included antibiotic therapy, surgical removal of the device and LAA surgical ligation in 3 patients (60%) [73, 74, 75], and antibiotic therapy alone in 2 (40%). Mitral valve replacement was also needed in one patient. One patient developed hemothorax, encephalopathy, respiratory failure requiring tracheostomy, and occlusive thrombosis of the right axillary vein after surgery [75]. Four patients (80%) [73, 75, 76, 77] were discharged alive, while the fifth died of cardiogenic shock (surgical case). Postoperatively, 1 patient (33%) [74] developed brainstem stroke that resolved within 24 h with residual generalized weakness and disability; another patient (not operated on) underwent lifelong suppressive antibiotic therapy [76].

In conclusion, LAAO-related IE is a rare yet highly morbid complication. According to the limited current evidence, systemic antibiotic therapy and surgical removal of the device are crucial for management.

### 3.4 Inter-Atrial Shunt Device 

Heart failure with preserved ejection fraction (HFpEF) constitutes more than half of heart failure cases, associates with poor prognosis and lacks approved and effective treatments [78]. Inter-atrial shunting, which allows for reverse blood flow from the left to the right side of the heart, was hypothesized to improve HFpEF symptoms by decreasing left atrial pressure without compromising left ventricular filling [79]. This was the basis for the creation of the transcatheter inter-atrial shunt device (IASD) [78, 79].

An early open-label clinical trial with 64 patients with HFpEF receiving IASD did not report any device-related infectious event at 6 months follow-up [78]. Similarly, and in a subsequent randomized, blinded and sham-controlled trial with 44 patients (22 with IASD), no device-associated infections were reported at 1 month [80]. In a more recent randomized controlled trial, 626 participants with HFpEF were enrolled and 314 received IASD, and results did not demonstrate any device infection, up to 12 months of follow-up [81]. These results were also similar to other studies [82, 83].

In summary, IASD-associated infections seem to be extremely rare, and no study demonstrated an infection so far. However, most available studies focused on the acute outcomes, and thus long-term follow-up data are awaited to determine the safety and efficacy of IASD [81].

## 4. Leadless Pacemaker 

**Fig. 2.**
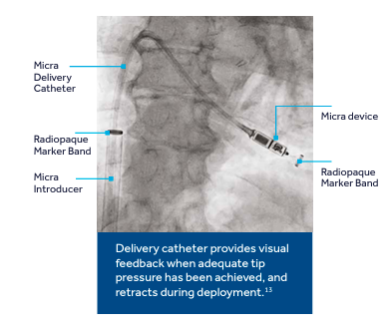
**Micra delivery system [84]**.

Leadless pacemakers (LPs) are new cardiac implantable electronic devices approved for the treatment of bradyarrhythmia in specific settings such as in patients with risk of transvenous device complications due to old age, significant comorbidities, or high infection risk [5]. Nanostim® LP (St. Jude Medical/Abbott, IL, USA) and Micra® Transcatheter LP (Medtronic, MN, USA) are the two available types of LPs (Fig. 1D), although only the latter is currently used. Both devices are implanted into the right ventricle (RV) using their own delivery system through the femoral vein (Fig. 2, Ref. [84]).

Two cases of definite LPs infections are reported in the literature. The first case was described by Koay *et al*. [85], and was an 80-year-old woman admitted with fever, chills, and rigors one month after Micra® implantation due to junctional bradycardia. Diagnosis was done by blood cultures, which grew methicillin resistant* S. aureus *(MRSA), and TEE, which revealed device vegetations. Treatment initially included targeted antibiotic therapy for one week, but due to persistently positive blood cultures, the device was later removed. Afterwards, the patient received 6 weeks of antibiotic treatment and the infection was resolved. A histopathological analysis of the device vegetation revealed findings consistent with infection (device culture not available) [85].

The second case was an 80-year-old man who developed pocket erosion, without evidence of blood stream infection, nine years after dual-chamber pacemaker (PM) implantation for sick sinus syndrome and atrioventricular block. Subsequently, the generator was removed leaving the leads behind, abandoned under the skin, and a Micra® was implanted into the right ventricle. One month later, the patient had recurrence of the erosion and cultures grew MRSA, with no vegetations visible on TEE. An emergency extraction of Micra® and the leads was done due to failure to respond to antibiotics, and culture of the device and leads grew MRSA. Treatment also included a 6 week course of antibiotics and, after 10 days of documented negative blood cultures, implantation of a cardiac resynchronization therapy device, with no recurrence of infection at nine-month follow up [86].

Initial studies with LPs did not detect any device infections on follow-up [87, 88], which was also the case in subsequent larger observational studies [89, 90, 91, 92]. LPs were safely implanted in high infection risk settings, as in patients with active infections—including CIED pocket infection—and positive blood cultures [93, 94]. Other settings where LP were apparently successfully used were patients who developed a serious infective event after other device implantation [95], patients on chronic hemodialysis [96], nonagenarians [97] or those with a heart transplant [98]. LPs implantation after cardiac implantable electronic device (CIED) infection, was successful and reduced recurrence rates [99, 100, 101, 102, 103]. Consistently, in the few studies comparing infective complications in LPs and PM patients, PM showed higher rates of these complications [104, 105, 106].

Several factors are thought to account for the safety of LPs. Some studies suggest that their small size (∼616 ${\mathrm{mm}}^{2}$ and ∼846 ${\mathrm{mm}}^{2}$, in Micra® and Nanostim®, respectively, compared to ∼3500 ${\mathrm{mm}}^{2}$ in PM), independence of pockets, and complete and fast encapsulation within an endothelial biomatrix, play an important role in their safety. Other alleged factors include the minimal handling of LPs during implantation, the high velocity and turbulence of blood flow in the RV, and the device constituents themselves (parylene-coated titanium and titanium vs titanium polyurethane and silicone in LPs and PM, respectively) [107]. A very likely additional factor is represented by the absence of any anatomical contiguity with the tricuspid valve leaflets, which generate turbulence and likely heamostasis system activation at the site of CIED lead passage through the annulus.

Some limitations of the reported studies with LPs include shorter time of follow-up compared to transvenous CIEDs [108]. In some reports, device culture was not documented after extraction, and device infection was often excluded solely based on visual inspection and/or no vegetation on TEE. Thus, it is not possible to exclude that some LPs infections were missed, implying more evidence is needed to ascertain the safety of LPs on longer term follow-up.

In conclusion, currently available evidence suggests that LPs are cardiac implantable electronic devices with a low risk of infection and can be considered very useful and safe especially in patients at high risk of infections, after CIED extraction for infection in PM-dependent subjects and when limited access to/patency of subclavian veins is an issue.

## 5. Newer Implantable Cardioverter-Defibrillators

### 5.1 S-ICD

S-ICD is a novel CIED used in patients with high risk of infection and an indication for ICD when pacing therapy for bradycardia support, cardiac resynchronization or anti-tachycardia pacing is not needed [2, 3, 109]. It is composed of a completely extrathoracic generator and a single subcutaneous lead.

As expected, none of the available studies showed IE related to S-ICD. However, early results from the EFFORTLESS S-ICD registry and S-ICD IDE study showed systemic infection rates of 2.4% and 5.7%, respectively [110, 111]. More specifically, the reported rates are more variable, as for instance in a study with 3717 consecutive S-ICD patients, only 3 infections were reported (0.08%) [112]; another study found an infection rate of 1.2% within 30 days post implant [113]. On long term follow-up, one study noticed an infection rate of 6.7% over 6 years, concentrating in the first month following implantation (37.5%), then at one year (37.5%), and more spread in the following five years (25%) [114].

Important information about infections in S-ICD carriers were obtained from studies comparing S-ICD and other types of intravascular defibrillators. Brouwer *et al*. [115] compared patients in SIMPLE and EFFORTLESS studies and found infections in 2.6% and 0.5% of patients with S-ICD and transvenous implantable cardioverter defibrillator (TV-ICD), respectively, but this apparent difference was not statistically significant. Moreover, and in a multicenter study, the adjusted infection rate of was 4.1% and 3.6% for S-ICD and TV-ICD, respectively, but nonlead-related (pocket) infections were more common in S-ICD (*p* = 0.047) [116], as also shown on long-term follow-up of more than four years [117]. Other devices compared to S-ICD included single chamber ICD and dual chamber ICD, and no difference in infection rates was observed [112].

One of the analyzed aspects is the safety of S-ICD in patients with active endovascular infections. In a study with patients who recently had their TV-ICD removed due to infection, 90 and 139 patients received S-ICD and TV-ICD, respectively, and were compared. After a median follow up of 17 months, one infection was reported in the S-ICD group, as compared to two in patients with TV-ICD, highlighting the safety of the S-ICD implantation after a previous TV-ICD infection [118]. Moreover, S-ICD were shown to be safe in immunocompromised patients, including those on hemodialysis [119].

In conclusion, the S-ICD is a novel device not free of infectious complications. Further investigation of risk factors for S-ICD infection is warranted. No IE cases were reported with S-ICD since it lacks the intravenous lead, used in TV-ICD. As most studies reported peak early infections, patients with S-ICDs should be closely assessed for infection at the generator pocket site when presenting with signs of systemic infection.

### 5.2 Extravascular Implantable Cardioverter-Defibrillator 

The extravascular implantable cardioverter-defibrillator (EV-ICD) is a novel device with a substernal lead placement, which like S-ICDs has the advantage of avoiding venous access and its associated complications [120]. However, unlike S-ICDs, EV-ICD has shorter leads due to its proximity to the heart, and thus overcomes some inherent limitations of S-ICDs, where longer leads in the latter prevent anti-tachycardia pacing (ATP) and require higher energy for defibrillation [121].

Currently, few feasibility studies are available in which the device was implanted and removed in the same procedure [122, 123, 124]. One chronic implantation study was available with EV-ICD. In this study, 20 patients were implanted, and only one reported superficial wound infection at the xiphoid incision site at 90 days follow up, which was treated with antibiotic therapy and apparently resolved without sequelae [125]. A trial with chronic implantation of EV-ICD is currently ongoing [126].

At present, available data are not adequate to evaluate the infectious risk of EV-ICD. Since the anterior mediastinum does not have adequate blood perfusion, infectious risk is important to properly explore, including acute bacterial mediastinitis, subcutaneous lead and pocket infections [121].

## 6. Cardiac Contractility Modulation

Cardiac contractility modulation (CCM) is a novel device-based modality developed for chronic heart failure treatment, and mainly functions by delivering non-excitatory signals during the absolute refractory period of the heart [127, 128, 129]. The device includes an impulse generator subcutaneously placed in the left or right pectoral region, connected by two transvenous leads to the right ventricular septum, with or without an additional lead to the right atrium (Fig. 1E) [130, 131, 132]. CCM was shown to increase left ventricular contractility and exercise tolerance in select advanced heart failure with reduced ejection fraction (HFrEF) patients, without increasing myocardial oxygen requirement [130, 133, 134].

In an early pilot study, thirteen patients with advanced HFrEF received a CCM device; after a mean of 7 months, only one patient (8%) experienced a pocket infection and was subsequently treated successfully with antibiotics [135]. A recent systematic review of four clinical trials found 9 cases of device pocket infection out of 469 included participants (2%), after a mean follow-up of six months [136]. One study with longer follow-up retrospectively included 54 HFrEF patients with CCM device and found 6 participants (11%) with device-related infection, over 7 years [137]. Most of these infections started with signs of pocket skin necrosis and advanced to bacterial infection, and caused death in half of affected patients [137]. Furthermore, a recent clinical trial (the FIX-HF-5C2 study) investigated the safety and efficacy of the two-lead system and reported one case of infection among 60 participants over a 6-month period [138]. Other studies with CCM did not show increased adverse events, including infections [134, 139, 140, 141, 142, 143].

Overall, CCM associated infections seem to be uncommon in the short term, and apparently readily treatable with no major serious sequelae. However, further investigation of the devices’ safety is warranted, since currently available studies are still limited, most notably regarding long-term outcomes (>6 months) [136]. In addition, differences in the methods of current studies were noted, including lacking blinding and/or sham procedures in some [136, 142]. Thus, we believe future studies must include proper control groups to better define the CCM-related infection risk. Likewise, most available studies do not thoroughly describe the infection, including microbiological culture results, clinical signs and symptoms and management course, which are key for infection risk investigation.

## 7. Conclusions and Future Outlook

Despite of their favorable cardiovascular safety profile, novel cardiac devices such as MitraClip, occluder devices, LPs, and S-ICD can undergo infection at virtually any time after implantation. As signs of device infection are non-specific and mostly include fever, this diagnosis should be ruled out whenever these patients present with non-organ specific signs of systemic infection. Although most patients did not suffer any long-lasting complication and were discharged alive after treatment, some suffered from complications such as stroke, and others died. Apart from the newer ICDs, cardiac modulation devices and inter-atrial shunts, new cardiac device infection mortality seems to overall overlap with classical prosthetic valve IE [144], and appears to be significantly less than Transcatheter Aortic Valve Implantation (TAVI)-related IE [145]. Further studies should investigate risk factors for the development of infections with these devices and compare medical and surgical management to improve the prognosis. Diffuse reporting of even single case reports appears crucial in light of the overall low rate of new cardiac device infections, so to allow systematic literature review and advancement of knowledge in this emerging field of cardiovascular medicine and clinical infectious disease.

We believe interventional cardiologists should be well aware of the infection risk associated with new cardiac device implant procedures since the initial placement. They should prescribe right away blood cultures whenever lone fever occurs in carriers of these devices. Infectious disease physicians should become acquainted with newer cardiac device structure and function to adequately treat these emerging infections.
